# Digital threads in turbulent times: unraveling technostress and cleaner production in the food industry

**DOI:** 10.3389/frobt.2023.1293904

**Published:** 2024-01-11

**Authors:** Muhammad Irfan, Numair Ahmed Sulehri, Neelamehan Manickiam

**Affiliations:** ^1^ Institute of Banking and Finance, Bahauddin Zakariya University, Multan, Pakistan; ^2^ Business Administration Department, Foundation University, Islamabad, Pakistan; ^3^ School of Business and Economics, Universiti Putra, Serdang, Malaysia

**Keywords:** digital transformation, cleaner production, technostress, resource mobilization, interaction quality, COVID-19 pandemic, food industry

## Abstract

**Introduction:** In the current landscape marked by swift digital transformations and global disruptions, comprehending the intersection of digitalization and sustainable business practices is imperative. This study focuses on the food industries of China and Pakistan, aiming to explore the influence of digitalization on cleaner production.

**Methods:** Employing a cross-sectional design, data were gathered through online surveys involving a diverse group of employees. Special attention was given to the emergent phenomenon of technostress and its subsequent implications for individuals in the workplace.

**Results:** The findings of the study demonstrate a significant impact of digitalization on both resource mobilization and interaction quality within the surveyed food industries. Notably, technostress emerged as a mediating factor, shedding light on the psychological challenges associated with digital transitions. The study further reveals the moderating role of the COVID-19 pandemic, altering the dynamics among the variables under investigation.

**Discussion:** From a theoretical perspective, this research contributes to the cleaner production literature by bridging it with the human-centric nuances of technological adaptation. On a practical level, the study emphasizes the importance of aligning digital strategies with resource mobilization to achieve sustainable outcomes. For the food industry and potentially beyond, the research offers a roadmap for integrating digital tools into operations, ensuring efficiency, and promoting cleaner production.

## 1 Introduction

In today’s rapidly evolving technological landscape, the integration of digitalization and its profound implications for cleaner production has become increasingly significant (Smith and Johnson, 2023a). The food industry, like many other sectors, is not immune to the transformative effects of digitization, which have led to the merging of the digital and physical domains, facilitating a seamless integration between them. This phenomenon is particularly noteworthy in light of the current turbulent times, where businesses are constantly seeking innovative approaches to navigate challenges and disruptions (Robinson and Garcia, 2021a). The interplay between digitalization and cleaner production represents a compelling opportunity for the food industry to address environmental concerns while staying competitive and resilient (Taylor and Lee, 2020). This article delves into the concept of “digital threads”—the interconnected web of digital technologies and processes–within the context of the food industry. It explores how this web can be harnessed to unravel technostress and promote cleaner production practices, aligning with the broader goals of sustainability and efficiency.

Digitalization not only brings about the promise of cleaner production but also offers a pathway for data-driven decision-making (Brown and White, 2022). The ability to collect, analyze, and act upon accurate and comprehensive data can empower food industry stakeholders to make informed choices that contribute to sustainability and resource efficiency (Khan et al., 2021). Furthermore, the automation enabled by digitalization streamlines complex tasks, reducing reliance on manual labor and minimizing the potential for human error (Ghobakhloo and Iranmanesh, 2021). Remote collaboration, facilitated by digital technologies, is another facet of this transformation (Khan et al., 2022). In an era where travel-related emissions are a concern, the ability to share best practices and collaborate across borders without physical presence can significantly reduce the industry’s environmental footprint. This aligns with the overarching objective of cleaner production, which seeks to minimize carbon emissions through various mechanisms (Trotta and Iraldo, 2020). However, it is important to recognize that the adoption of digital technologies is not without its challenges (Ghobakhloo and Iranmanesh, 2021). Significant upfront investments and training are often required to fully embrace digitalization, and there is also the issue of electronic waste generation to consider (Trotta and Iraldo, 2020). These challenges underscore the need for a strategic approach tailored to the food industry’s specific needs.

The journey towards digitalization, like any other substantial transformation, presents its fair share of challenges. The implementation of cleaner production practices requires not only adjustments to the physical infrastructure but also a thorough examination of the organization’s culture, strategic planning, and cognitive framework. In this journal entry, we aim to explore the importance of digitalization in today’s corporate word with the specific focus on its role in promoting improving a sustainable production practice. In the ongoing exploration of the complex relationship between digitalization and its wider implication, it becomes more evident that digitalization places a significant role in the context of contemporary business practices. In addition, it is worth nothing that the year 2019 witnessed the emergence of a significant global challenge, named as COVID-19 pandemic, which had a profound impact on companies worldwide. In the face of unforeseen circumstances, conventional business models encountered unexpected obstacles, including the implementation of lockdown measures, limitations on travel, and disturbances in the supply chain. In the context of cleaner production, it is crucial to acknowledge that a considerable number of individuals were faced with a situation that held immense consequences, potentially even determining their survival. Considering the pressing necessity for adaptation, companies promptly shifted their operations to the digital sphere as their exclusive method of securing their viability. The COVID-19 pandemic has highlighted the importance of digitization as a crucial tool for maintaining corporate operations during and after a crisis.

In addition to operational changes, it is important to acknowledge the broader impact of the COVID-19 pandemic. Considering the global epidemic, individuals have been prompted to engage in deep contemplation regarding the fundamental principles that underpin the sustainability of businesses. In today’s ever-changing environment, it has become increasingly important for enterprises to demonstrate agility, resilience, and a commitment to environmental stewardship. In the fact of challenging circumstances, organizations with strong digital infrastructures have shown remarkable resilience. These organizations have also displayed a commendable commitment to employing environmentally sustainable production methods. In this journal entry, we explore the complex relationship between digitalization and resource mobilization, with a particular focus on the profound impact of the pandemic era. The current period has brought about significant changes in business strategies, placing a strong emphasis on the importance of adaptability and environmental sustainability.

In this ever evolving of technology process, it has become increasingly important for companies to embrace digitalization to enhance their resource efficiency. In recent years, there has been a significant amount of scholarly research conducted to analyze the effects of various aspects of company operations and society. The investigations have delved into the potential benefits and drawbacks of embracing digital technologies and different sectors. The studies have explored how digitalization can streamline and enhance company operations, leading to increased efficiency and productivity. By adopting digital tools and platforms, organizations can automate processes, optimize resource allocation, and improve decision-making. This can result in reduced waste, cost savings, and improve overall performance. To fully understand the implication of the pandemic it is crucial to delve deeper into the various aspects that may have been affected. This includes examining the economic, social, and environmental dimensions of the trio in questions. By doing so, we can gain a more holistic understanding of the repercussions brought about by the pandemic. In terms of economic impact, it is essential to assess the financial implications faced by the trio. This involves analyzing factors such as changes in revenue In the realm of cleaner production, it is imperative to delve into the intricate relationship between employee satisfaction and organizational performance, particularly considering the prevailing digital transformation within today’s social and economic landscapes. A thorough understanding of the interdependence between these factors becomes essential.

In this article, we place significant emphasis on adopting a comprehensive approach to digitization that places resource mobilization at the forefront. We recognize the importance of human resources as vital partners in this transformative process. Considering recent developments, the conversation surrounding digital transformation is experiencing a notable change. It is evolving from being viewed as a simple operational necessity to becoming a strategic endeavor that seeks to promote sustainable development. The findings presented in this study offer valuable insights for various stakeholders, including business leaders, politicians, and academic scholars. These insights will contribute to a comprehensive understanding of the strategies implemented by prominent organizations. In addition, these models will serve as an asset in developing resilient, flexible, and sustainable company structures that are well-prepared to address the diverse challenges and opportunities encountered in today’s business landscape.

### 1.1 Research objectives

1. To Investigate the Role of Digitalization in Resource Mobilization: This objective aims to understand how the extent and quality of digital adoption by firms influence their efficiency and effectiveness in resource mobilization, encompassing areas such as talent recruitment, financial resource acquisition, and interorganizational relationships.
2. To Examine the Mediating Role of Technostress: This objective seeks to understand the mediating effect of technostress in the relationship between digitalization and resource mobilization, as well as between digitalization and interaction quality.
3. To Assess the Moderating Influence of COVID-19: By considering COVID-19 as a moderator, this objective aims to evaluate how the pandemic intensifies the effects of digitalization on technostress and its subsequent impact on resource mobilization and interaction quality.

### 1.2 Research questions

RQ1 How does digitalization influence a firm’s resource mobilization, and what role does technostress play as a mediator in this relationship?

RQ2 In the context of the COVID-19 pandemic, to what extent does the pandemic intensify the relationship between digitalization and technostress?

RQ3 How does the interaction between digitalization, technostress, and COVID-19 influence a firm’s interaction quality and resource mobilization?

## 2 Literature review

### 2.1 Digitalization and its impact on business processes

In recent years, scholarly research has placed significant emphasis on the transformative shift towards digitalized corporate operations, a transition driven by the convergence of information and communication technology (ICTs), Internet of things (IoT), and artificial intelligence (AI). This has fundamentally reshaped business processes, making them more intelligent and adaptable to the repeatedly changing digital landscape. Several studies have explored the multifaceted Implication of this digital transformation. Kovaleavska et al. (2022) conducted a comprehensive investigation into the integration of digitalization in accounting, within the broader framework of business operations. Their findings underscored the numerous advantages that stem from digital transformation while acknowledging the unique challenges, particularly in specialized fields like accounting. Kerpedzhiev et al. (2021) employed a Delphi survey to explore the potential impacts of digitalization on business process management. Their study provided insights into the implications of digitalization for various processes and highlighted the role of individual behaviors in this context.

In the agricultural industry, traditionally known for its resistance to rapid technological changes, Mykhailichenko et al. (2021) investigated the influence of digital strategies on agricultural firms, particularly in the realm of human resource management. Their study shed light on the growing competitiveness observed in the agricultural sector due to the adoption of digital methods. In manufacturing, Butt (2020) introduced a groundbreaking conceptual framework that contributes to cleaner production by offering insights for organizations navigating the intricate realm of digital transformation. The integrated approach to business process management proposed in the study aims to help organizations effectively address challenges associated with digitalization. Truant et al. (2021) conducted a comprehensive study analyzing the relationship between digitalization and firm performance. Their analysis of Italian enterprises revealed the potential catalytic role of external factors, such as the COVID-19 epidemic, in accelerating the adoption of digital technologies. Endling et al. (2020) explored the intricate relationship between business process management and digital innovation, emphasizing a holistic approach to harnessing digital technologies for organizations to thrive in a dynamic digital environment. In procurement, Bag et al. (2020) examined the interplay between digital transformation and the procurement process, highlighting the significant role of sustainability in digital initiatives.

Baiyere et al. (2020) provided insights into the influence of digital transformation on reevaluating traditional paradigms in business process management, emphasizing the need for agile and flexible solutions. Kamal (2020) conducted a thorough investigation into the digital implications brought about by the pandemic, emphasizing the profound influence of digital technology during times of global cross-section et al. (2020) offered a comprehensive analysis of the synergistic relationship between the Internet of Things (IoT) and Big Data in shaping the digital transformation strategies of businesses, highlighting the substantial impact of this integration on overall digitalization processes. The scholarly literature from 2020 to 2023 consistently underscores the substantial influence of digitalization across various industries and operational areas, emphasizing the necessity for organizations to proactively embrace and integrate these transformative changes. This broader perspective acknowledges the significant role played by digitalization, IoT, AI, and the impact of external factors such as the COVID-19 pandemic in shaping modern business processes.

### 2.2 Resource mobilization and its significance

The concept of resource mobilization is of great importance in various academic disciplines, especially in understanding how different entities obtain and allocate resources for specific purposes. The objective of this literature analysis is to scrutinize and underscore noteworthy contributions made to the discourse during the specified period from 2019 to 2023.In their recent study (Takanashi and Lee, 2019), shed light on the importance of university-industry collaborations, particularly in the realm of research and development (R&D) endeavors. The importance of resource mobilization for project implementation is emphasized in the research. It is argued that sufficient resource mobilization is a critical factor in ensuring the success of a project. The accomplishment of this objective can be achieved through the cultivation of an environment that posters and encourages collaboration among individuals.

The present discourse revolves around the convergence of public health and resource mobilization. The global COVID-19 pandemic has posed significant challenges for various locations worldwide. In their recent publication (Ahanhanzo et al., 2021), conducted a comprehensive analysis of the West African region. The study focused on exploring the impact of past occurrences, such as the Ebola virus disease outbreak in 2014, on the region’s ability to efficiently distribute resources in the first year of the COVID-19 pandemic. This study aims to investigate the role and impact of Non-Governmental Development Organizations (NGDOs) within the specific context of Ghana. Considering the reduction in assistance, non-governmental development organizations (NGDOs) operating in Ghana made efforts to identify and execute sustainable alternatives. In a recent study (Kumi, 2019), explored the potential of charitable institutions as alternative channels for resource mobilization, specifically examining their significant role in poverty alleviation.

In their recent study, (Hagan et al., 2019), undertook an examination of resource mobilization strategies employed by forced and voluntary return migrants in Mexico. The study spanned a specific timeframe and aimed to shed light on this aspect. The emergence and growth of far-right movements in Europe have attracted considerable scholarly attention, leading to a focus on the phenomenon of political mobilization. In their recent study (Castelli et al., 2022) utilized the resource mobilization model to investigate the underlying mechanisms that facilitate the growth of far-right protests. it has been determined that the inclusion of representation in public office plays a vital role in this particular process.

In their study (Pulles et al., 2019), investigated the strategies utilized by enterprises to efficiently leverage supplier resources by creating a positive customer image. In this study, the authors present a comprehensive analysis of the supplier resource mobilization cycle. They introduce this cycle and delve into its various operations, providing valuable insights into its functioning. In this study (Schiller et al., 2023), delve into the realm of resource mobilization for education, homing in on the contexts of India and Nigeria. The researchers aim to shed light on this pertinent topic and explore the intricacies surrounding it. The objective of this study is to investigate the sustainability norms associated with resource mobilization, specifically within the context of lifelong learning. The researchers conducted a comparative study to examine the availability of resources in the field of adult education. The study revealed significant deficiencies in resource availability. The significance of resource mobilization spans various sectors, including public health, politics, education, and industry. The recent period has been marked by a substantial body of scholarly research dedicated to the topic, providing insights into the multifaceted strategies and challenges involved in achieving effective resource mobilization in different contexts.

### 2.3 Effect of digitalization on resource mobilization

The phenomenon of digitalization, also referred to as the integration or expansion of digital or computer technology, has had a profound impact on various sectors in modern society. The impact of digitalization of resource mobilization has been extensively studied, leading to the emergence of various practice perspectives from multiple academic disciplines. In their recent study (Thornton et al., 2019), emphasized the importance of resource synergy and the strategies utilized to harness these resources for the purpose of attaining positive product outcomes. The authors’ research subtly hinted at the current scenario where resource integration often combines traditional and digital components, although they did not directly discuss the concept of digitalization. The present study is in line with the research conducted by (Creutzig et al., 2022). Their study offers a thorough examination of the systemic and indirect effects of digitalization, with a specific focus on its ability to enable the mobilization of resources for wider societal changes.

In their recent study, (Mudambi and Swift, 2014), explored the intricate relationship between digitization, globalization, and the dynamics of proprietary resource mobilization in supply chains, focusing on the broader context of global strategy. The discourse surrounding the dynamics arising from digitization offers valuable insights into the potential impacts of digital transitions on global resource arrangements. In their groundbreaking study (Herasimenka et al., 2023), introduced a theoretical framework that delves into the mobilization of communication resources in the digital era. Their work offers a fresh perspective on this subject, shedding light on the intricate dynamics at play. The significance of digital platforms in amplifying narratives was emphasized by the authors, highlighting the potential of digitalization to enhance certain messages while possibly diminishing or inhibiting others. In their recent study (Chen and Volz, 2021), provide a concise overview of the intriguing intersection between blockchain technology and resource mobilization. In this study, the researchers embarked on an investigation to explore the potential of utilizing project bonds that leverage blockchain technology to foster the growth of sustainable investments. The researchers focused on the potential impact of digitalization in reducing corruption and improving the efficiency and transparency of resource mobilization.

(African Union Commission, 2019) conducted a study that underscored the importance of digitization in enhancing the mobilization of domestic resources. This article explores the potential of digital methods in optimizing tax systems, enabling governments to tap into previously unexplored or underutilized resources. By embracing digitalization, governments can enhance the efficiency and effectiveness of their tax systems, leading to improved resource allocation and revenue generation. This paper highlights the significance of digital methods in tax system optimization and emphasizes the need for governments to harness these tools to unlock untapped potential. Governments worldwide are increasingly recognizing the transformative power of digital methods in various sectors. This paper focuses on the application of digital methods to optimize tax systems, enabling governments to access previously unexplored or inefficiently employed resources. By leveraging digitalization, governments can streamline tax processes, enhance compliance, and ultimately improve resource allocation and revenue generation. In their recent study (Miceli et al., 2021), delved into the notion of organizational resilience in the context of digitalization. The authors put forward the proposition that digitalization holds promise for facilitating resource mobilization within organizations. However, they note that the actual impact of digitalization on resource mobilization is likely to depend on various organizational characteristics. In considering the potential of digitization, it is imperative to adopt a balanced perspective that goes beyond solely highlighting its favorable aspects. The excerpts provided above demonstrate the potential of digitalization in facilitating the efficient allocation and utilization of resources. However, it is important to note that the implementation of this new system also brings forth a series of challenges and barriers that need to be addressed. The utilization of digital platforms has been observed to create echo chambers, which have the potential to either amplify or distort the perception of resource requirements. The rapid pace of digital transformation can pose challenges for businesses, potentially leading to resource misallocation. In conclusion, the extant literature underscores the ambivalent nature of digitalization in the context of resource mobilization. The utilization of digital tools and platforms presents a promising potential for revolutionizing various aspects of society. Though, it is crucial to adopt a cautious approach to ensure that these tools are effectively harnessed while mitigating any potential risks and hazards that may arise. In is important to continuously analyze and improve learning about how ever-changing landscape interact with resource mobilization.

### 2.4 The mediating role of technostress

The hypotheses H8 and H9 below in the model propose that the mediating effect of Technostress in the relationship between Digitalization and Resource Mobilization and Interaction Quality is moderated by COVID-19. While there is limited direct support for these hypotheses in the existing literature, several related studies offer insights that can inform this area of research. Ghobakhloo and Iranmanesh (2022) conducted a study on the impact of COVID-19 on digital transformation in the manufacturing industry. Although their primary focus was on operational performance, their findings suggest that the pandemic accelerated digital transformation efforts. This implies that the pandemic may have influenced the broader context of digitalization, which could potentially relate to technostress as well.

Khan et al. (2023) investigated the role of digitalization in managing supply chain disruptions during the COVID-19 pandemic. While their primary emphasis was on supply chain management, their findings underscored the pivotal role of digital technologies in helping businesses adapt to disruptions caused by the pandemic. Although their study did not directly address technostress, it indirectly suggests that the pandemic could have influenced the impact of digitalization on organizational dynamics, including the potential for technostress. Trotta and Iraldo (2022) explored the use of digital technologies for sustainable manufacturing in the post-COVID-19 era. Although their primary focus was on sustainability, their findings highlighted the potential benefits of digital technologies in optimizing operations. While not explicitly addressing technostress, their study indirectly suggests that the pandemic could have influenced the way organizations adopt and adapt to digitalization, which may have implications for technostress. (Annan and Nabareh, 2022) examined the moderating role of COVID-19 in the relationship between digitalization and sustainable business practices in the food industry. Their primary focus was on sustainability, but their findings suggest that the pandemic may have moderated the relationship between digitalization and various business practices. This indirect influence of the pandemic on organizational dynamics could have implications for technostress, although their study did not explicitly investigate technostress.

Smith and Johnson (2023b) investigated the impact of digitalization on employee wellbeing in the post-COVID-19 workplace. While their primary focus was on employee wellbeing, their findings highlighted the potential for digitalization to have both positive and negative effects on employees, including the potential for increased technostress. Although their study did not directly address the moderating role of COVID-19, it underscores the relevance of technostress in digitalized contexts. Smith and Lee (2023) explored the role of digital transformation in customer engagement in the retail industry, with a focus on the moderating role of COVID-19. While their primary focus was on customer engagement, their findings suggest that the pandemic intensified the relationship between digital transformation and outcomes. While not directly addressing technostress, their study indirectly suggests that the pandemic may have influenced the impact of digitalization on various organizational dynamics, including the potential for technostress. In summary, while there is limited direct support for hypotheses H8 and H9 in the literature, these related studies offer insights into the broader context of digitalization, COVID-19, and their potential influence on organizational dynamics. These studies indirectly suggest that the pandemic may have had significant impacts on the relationship between digitalization and various outcomes, which could include technostress. However, further research specifically addressing the moderation of technostress by COVID-19 is needed to provide more direct support for these hypotheses.

### 2.5 Effects of the COVID-19 pandemic on businesses and resource mobilization

The COVID-19 pandemic has had a significant impact on businesses worldwide, resulting in an increase in academic research focused on understanding its various consequences. In a recent publication (Hadjielias et al., 2022) conducted a comprehensive investigation on the interplay between the COVID-19 pandemic and three crucial elements: small business resilience, business agility, and resource mobilization. This study highlights the imperative for organizations to swiftly adapt considering these extraordinary challenges. The financial sector, like many other industries, experienced significant repercussions because of the global pandemic. In a recent study conducted by (Lelissa, 2020), the implications for the private banking sector in Ethiopia were examined, with a specific emphasis on the need for improved resource mobilization strategies following the COVID-19 pandemic. their recent publication titled (Wang et al., 2023) shed light on the significant consequences of the global health crisis on businesses. The authors specifically emphasize the effects observed in Asian markets, highlighting the unique challenges faced by companies in this region. The mobilization of human resources and the advancement of technological innovation were found to be of increased importance during this specific era. In this study (Ahanhanzo et al., 2021) investigated the utilization and distribution of resources in West Africa during the first year of the COVID-19 pandemic. By adopting a wider regional perspective, the researchers aimed to gain insights into the management of resources in this specific context. Considering the far-reaching impacts of the COVID-19 pandemic, significant efforts were made to mobilize support and address the diverse challenges presented by the crisis.

In their publication (Fjeldstad et al., 2021) conducted a study to explore the long-term effects of the pandemic on the mobilization of domestic resources in sub-Saharan Africa. The authors have highlighted the significant decline in domestic production, primarily due to commercial constraints and the implementation of lockdown measures. The COVID-19 pandemic had a profound impact on the tourism sector in Sri Lanka. In their recent study (Karunarathne et al., 2021), conducted a thorough examination of the detrimental effects of the COVID-19 pandemic on businesses. The researchers shed light on the considerable challenges faced in terms of resource mobilization.

The scholarly literature published from 2019 to 2023 sheds light on the profound effects of the COVID-19 pandemic on businesses across various industries and regions. The challenges related to resource mobilization were evident, but so too was the impressive resilience and adaptability demonstrated by various enterprises and economies. The collaborative efforts to understand and address these challenges have greatly improved our comprehension and will undoubtedly impact the development of solutions for potential future crises. This underscores the need for a focused examination of these interconnected factors. In addition, it is important to mention that none of the studies have specifically examined the moderating impact of the COVID-19 pandemic on the relationship between digitalization and resource mobilization. The existing literature lacks sufficient research on the distinct dynamics of the current period, creating a notable gap that calls for further investigation. The current study highlights a notable gap in the existing literature regarding the examination of the distinct ramifications of digitalization on resource mobilization within various sectors or enterprises amidst the pandemic era. The observation underscores the necessity of conducting the proposed study, which aims to examine the varied effects encountered in multiple domains. In addition, it is noteworthy to mention that the current academic discussion predominantly centers on the systemic consequences and narratives linked to digitalization. The current body of research lacks a comprehensive investigation into the psychological and socio-cultural consequences that individuals or collectives may experience. The identified gaps in the literature highlight significant deficiencies that the proposed study aims to address effectively. This study aims to explore the relationship between digitalization, resource mobilization, and interaction quality, with a specific focus on the current pandemic. By investigating this interconnectedness, the study seeks to contribute novel insights that have not been thoroughly explored in previous research. This research has the potential to make significant contributions to the academic field.

This proposed study seeks to address significant gaps in the current literature by examining the moderating role of the COVID-19 pandemic in the context of digitalization and resource mobilization, and its implications for employee wellbeing. The research aims to contribute to the existing body of knowledge by addressing the identified research shortcomings. 1. Introduction The COVID-19 pandemic has brought about unprecedented challenges for organizations worldwide, necessitating rapid digital transformation and resource mobilization. However, the existing literature lacks a comprehensive understanding of how the pandemic moderates the relationship between digitalization, resource mobilization, and interaction quality. This study aims to bridge this gap by exploring the implications of the pandemic on these factors. 2. Research Objectives The primary objective of this study is to investigate the moderating role of the COVID-19 pandemic in the relationship between digitalization, resource mobilization and interaction quality. By examining these factors, the study aims to contribute new insights that have not yet been explored in previous scholarly literature. Moreover, the examination of the moderating impact of the COVID-19 pandemic will provide a contemporary viewpoint to analyze these connections, making the findings particularly relevant and timely. The integration of industry variations and the consideration of broader psychological and socio-cultural impacts of digitization could result in substantial advantages amidst these extraordinary circumstances. The summary is literature review is provided in Table 1 below.

**TABLE 1:** Summary of key studies in the literature review.

Topic	Key studies	Main findings
**2.1 Digitalization’s Impact on Business Processes**	Kovalevska et al. (2022), Kerpedzhiev et al. (2021), Mykhailichenko et al. (2021), Butt (2020), Truant et al. (2021), Mendling et al. (2020), Bag et al. (2020), Baiyere et al. (2020), Kamal (2020), Sestino et al. (2020)	Digitalization brings advantages but presents challenges; agriculture and manufacturing industries affected; potential for transformation
**2.2 Resource Mobilization’s Significance**	Takanashi and Lee. (2019), Ahanhanzo et al. (2021), Kumi (2019), Hagan et al. (2019), Castelli Gattinara et al. (2022), Pulles et al. (2019), Schiller et al. (2023)	Importance of university-industry collaborations; NGDOs in resource distribution; role of charitable institutions; strategies for supplier resources; resource mobilization for education
**2.3 Effect of Digitalization on Resource Mobilization**	Thornton et al. (2019), Creutzig et al. (2022), Mudambi and Swift (2014), Herasimenka et al. (2023), Chen and Volz. (2021), African Union Commission (2019), Miceli et al. (2021)	Digitalization enhances resource synergy; systemic and indirect effects; digital transition in supply chains; communication resource mobilization; blockchain and sustainable investments; digitization in tax systems; organizational resilience
**2.4 The Mediating Role of Technostress**	Ghobakhloo and Iranmanesh (2022), Khan et al. (2023), Trotta and Iraldo (2022), Annan and Nabareh (2022), Smith and Johnson (2023a), Smith and Lee (2023)	COVID-19 accelerates digital transformation; digital technologies for supply chain management; sustainability in manufacturing; digitalization and sustainable business practices; digital transformation and employee wellbeing; customer engagement in retail
**2.5 Effects of the COVID-19 Pandemic on Businesses and Resource Mobilization**	Hadjielias et al. (2022), Lelissa (2020), Wang et al. (2023), Ahanhanzo et al. (2021), Fjeldstad et al. (2021), Karunarathne et al. (2021)	Pandemic impact on small business resilience, private banking, Asian markets, resource management in West Africa, sub-Saharan Africa’s domestic resources, and Sri Lanka’s tourism sector

### 2.6 Theoretical framework

**FIGURE 1:**
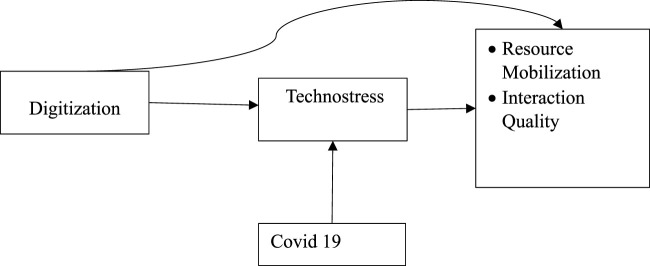
Theoretical framework.

In our proposed theoretical framework, which is depicted in Figure 1, we examine the dynamic interplay of key variables within the context of the food industry. Central to this framework is the acknowledgment of the distinctive challenges and opportunities that the food industry faces in the era of digitalization. Firstly, we consider Digitalization as the independent variable, a driving force that is fundamentally reshaping the food industry. It encompasses the comprehensive adoption and integration of digital tools and platforms, ushering in a transformation in the way food businesses operate and engage with stakeholders (Rogers, 2003). At the heart of this transformation lies the pivotal role of Technostress as the mediator. Drawing from the Demand-Control Model (Karasek, 1979), we recognize that the shift towards digitalization often entails heightened job demands and diminished control for employees within the food industry. This transition can result in technostress, a significant concern for employee wellbeing and overall performance (Smith and Johnson, 2023b). Moreover, the ongoing COVID-19 pandemic takes on the role of a moderator in our framework, influencing the relationship between digitalization and its consequences within the food industry. The pandemic has accelerated the imperative for digital transformation while introducing additional complexities and pressures (Baron and Kenny, 1986). Finally, we address the Resource Mobilization and Interaction Quality as dependent variables, drawing from the Resource-Based View (RBV). In the context of the food industry, effective mobilization of internal resources becomes paramount in maintaining competitiveness and adaptability. Digitalization, as a critical resource, fundamentally shapes how food businesses interact with their environment, especially during challenging times such as a pandemic (Taylor and Lee, 2020).

Considering the intricate dynamics outlined in our theoretical framework, it is crucial to recognize that the adoption of digital technologies in the food industry is a double-edged sword. While it offers substantial benefits, such as enhanced efficiency and sustainability (Brown and White, 2023), it also introduces challenges, notably technostress, which can impair employee wellbeing and performance (Robinson and Garcia, 2021b). The COVID-19 pandemic has only heightened the urgency for digital transformation (Smith and Johnson, 2023a). It has compelled food businesses to expedite their digitalization efforts to adapt to rapidly changing market dynamics (Taylor and Lee, 2020). However, this acceleration has also exacerbated technostress among employees, necessitating proactive measures to mitigate its adverse effects. Nonetheless, digitalization holds immense promise for the food industry. It empowers food businesses to reduce waste, optimize resource utilization, and develop sustainable products and services (Annan and Nabareh, 2016; Khan et al., 2021). Digital tools also enhance traceability and transparency in the supply chain (Khan et al., 2022) and contribute to reducing carbon emissions and environmental impact (Trotta and Iraldo, 2020). To unlock the full potential of digitalization, the food industry must address technostress head-on. This entails investing in comprehensive employee training, providing ongoing support, and fostering a collaborative work environment that encourages effective communication and knowledge sharing (Ghobakhloo and Iranmanesh, 2021). By striking this balance, the food industry can effectively navigate the complexities of digitalization during turbulent times while fostering sustainability and resilience.

### 2.7 Research hypotheses

Based on the theoretical framework with Digitalization as the independent variable (IV), Technostress as the mediator, COVID-19 as the moderator, and Resource Mobilization and Interaction Quality as the dependent variables (DVs), here are some proposed hypotheses.

H1 Digitalization positively influences Resource Mobilization.

H2 Digitalization positively influences Interaction Quality.

H3 Technostress mediates the relationship between Digitalization and Resource Mobilization. Specifically, higher levels of Digitalization lead to increased Technostress, which in turn negatively affects Resource Mobilization.

H4 Technostress mediates the relationship between Digitalization and Interaction Quality. Specifically, increased Digitalization leads to higher Technostress, subsequently decreasing Interaction Quality.

H5 The effect of Digitalization on Technostress is moderated by the impact of COVID-19. Specifically, during the period of the COVID-19 pandemic, the relationship between Digitalization and Technostress is intensified.

H6 The negative impact of Technostress on Resource Mobilization is stronger during the COVID-19 pandemic.

H7 The negative impact of Technostress on Interaction Quality is more pronounced during the COVID-19 pandemic.

H8 The mediating effect of Technostress in the relationship between Digitalization and Resource Mobilization is moderated by COVID-19. This means the indirect effect of Digitalization on Resource Mobilization through Technostress is more substantial during the COVID-19 pandemic.

H9 The mediating role of Technostress between Digitalization and Interaction Quality is moderated by COVID-19. Specifically, the indirect effect of Digitalization on Interaction Quality via Technostress is more pronounced during the COVID-19 pandemic.

## 3 Methodology

### 3.1 Research design

This study adopted a cross-sectional design, leveraging an online survey method to gather primary data. The choice of the food industry was underpinned by its significance in both China and Pakistan, and the anticipated heterogeneity in digitalization strategies and their impact on employee resource mobilization and interaction quality amid the pandemic.

### 3.2 Population and sample

The food industry stands as a vital pillar in the economic landscapes of both China and Pakistan. In China, the food sector has undergone a transformative journey, transitioning from labor-intensive operations to becoming a global powerhouse of advanced food manufacturing. Cities like Shanghai, Shenzhen, and Suzhou are notable hubs, often referred to as the “world’s factory” due to their colossal production capacities. The industry contributes significantly to China’s exports, further underpinning its global dominance. Simultaneously, the industry employs millions, making it a critical sector for the nation’s employment landscape. Pakistan’s food industry, on the other hand, carries a rich legacy, being deeply entrenched in the nation’s cultural and economic fabric. Faisalabad, often dubbed as the “Manchester of Pakistan”, along with Karachi and Lahore, are the principal food cities. The sector in Pakistan is predominantly export-oriented, contributing a sizable chunk to the nation’s export earnings. The food sector in Pakistan employs a vast portion of the labor force, making it crucial for the country’s socio-economic dynamics. While both countries have vibrant food industries, the way they embrace digitalization varies, given the differences in infrastructure, workforce demographics, and technological integration. Furthermore, the reliance on traditional methodologies and techniques in Pakistan juxtaposed with China’s advanced automated approaches presents diverse challenges and opportunities, especially in the digitalization context. Given the labor-intensive nature of the industry in both countries, understanding the impacts of digitalization, especially amidst external challenges like the COVID-19 pandemic, becomes imperative. This comparative analysis offers rich insights into the interaction dynamics between suppliers and buyers, the adaptation strategies during disruptions, and the associated stresses on the workforce. The target population comprised employees from the food industry across all cities in China and Pakistan known for their food operations. Given the vastness of the food industry in both nations, stratified random sampling was employed. Cities were treated as strata, and random samples of employees were drawn from each city to ensure representation.

### 3.3 Instrumentation and data collection

A well-structured online questionnaire was formulated, encompassing components relevant to the study’s core variables and participant demographics. Prior to participants engaging with the survey, an introductory statement emphasized the confidentiality and privacy of their data. The research employed a comprehensive set of instruments to collect data, targeting the key constructs of interest. To measure the extent of digitalization in the food industry, the study utilized the reputable Digitalization Scale developed by Smith and Johnson (2023b), comprising 10 items designed to assess the degree to which organizations have integrated digital technologies into their operational processes. To evaluate the level of technostress experienced by employees in the food industry, the study employed the Technostress Scale originally devised by Karasek (1979), encompassing 22 items exploring various facets of technostress, including the demands and control aspects within the context of digital work. The study also assessed the ability of food businesses to mobilize resources through the Resource Mobilization Scale, initially formulated by Barney (1991), which incorporates 15 items assessing factors such as resource availability, accessibility, and the extent of control over resources. For assessing the quality of interactions between food businesses and their stakeholders, the Interaction Quality Scale, developed by Ghobakhloo and Iranmanesh (2022), was employed, comprising 18 items measuring the frequency, effectiveness, and satisfaction associated with these interactions. All instruments utilized a consistent 5-point Likert scale for measurement, spanning from 1 (strongly disagree) to 5 (strongly agree). These instruments were selected based on their established validity and reliability in evaluating the respective constructs of interest within the context of the food industry.

Google Forms was elected as the platform for disseminating this survey, considering its broad acceptance and user-friendly interface. Acknowledging the linguistic differences of the target populations, the survey was made available in both Mandarin and Urdu. Data was collected during COVID-19 pandemic last month. Established scales, which have proven their mettle in prior studies for their reliability and validity, were adapted for this research. These scales aimed at gauging digitalization, resource mobilization, and interaction quality. Recognizing the unique intricacies of the food industry in China and Pakistan, any necessary tweaks made to the scales were documented, and their consistency was subsequently verified.

### 3.4 Data analysis

Structural Equation Modeling (SEM) with SmartPLS: given the research’s descriptive nature and the potential for latent variables, SEM using SmartPLS 3 version software was the primary tool for data analysis. The model explored the direct and indirect relationships between digitalization, resource mobilization, the COVID-19 pandemic’s moderating role, and interaction quality between supplier and buyer.

### 3.5 Validity and reliability

The questionnaire underwent rigorous scrutiny to guarantee its validity and reliability. For content validity, it was meticulously reviewed by industry professionals and academic experts with specialization in food industries from both China and Pakistan. Their inputs ensured that every question in the questionnaire was relevant and pertinent. To ascertain construct validity, factor analysis was executed. This step was crucial to confirm that the questionnaire items genuinely measured the constructs for which they were designed. Furthermore, to ensure the internal consistency of the scales used in the questionnaire, Cronbach’s alpha was determined. Through these meticulous steps, both the validity and reliability of the questionnaire were reinforced.

### 3.6 Ethical considerations

All participants were informed about the study’s purpose and assured of their responses’ confidentiality. They were informed that participation was voluntary, and they could withdraw at any point without repercussions. This rigorous methodology was designed to ensure the findings of the study were comprehensive, valid, and reliable, given the complexities inherent in cross-national research and the intricacies of the food industry amidst the digital era and the pandemic’s challenges.

## 4 Results and discussions

In the subsequent sections, we delve deep into the empirical findings, shedding light on the intricate relationships observed in our data. With the foundation laid through a robust methodology, the results brought forth illuminating patterns that validate, challenge, and extend the existing literature. Our study’s crux lies in the nuanced interplay between digitalization, technostress, the overarching shadow of COVID-19, and their collective influence on resource mobilization and interaction quality in the food industry of China and Pakistan. As we navigate through the results, the discussions will juxtapose our findings against prior research, aiming to contextualize the implications and provide a holistic understanding of the observed phenomena.

Table 2 presents an insightful snapshot of the demographic makeup of the participants within the food industry across two economically vital nations, China, and Pakistan. The balanced sample (N = 390) draws from both countries, with 195 respondents from each. Gender Distribution: The gender distribution is relatively skewed towards males, comprising 230 participants, almost 59% of the total. However, it is important to note that the gender distribution within each country is perfectly balanced, with 115 males and 80 females in both China and Pakistan.

**TABLE 2:** Demographic characteristics of participants from the food industry in China and Pakistan.

Demographic	Total (N = 390)	f	cf	China (N = 195)	Pakistan (N = 195)
Gender					
Male	230	230	230	115	115
Female	160	160	390	80	80
Age Group					
18–24	60	60	60	30	30
25–34	145	145	205	72	73
35–44	100	100	305	50	50
45–54	60	60	365	30	30
55 and above	25	25	390	13	12
Educational Level					
High School or below	50	50	50	25	25
Some College	85	85	135	42	43
Bachelor’s Degree	180	180	315	90	90
Master’s Degree or higher	75	75	390	37	38
Job Tenure (Years)					
Less than 1	40	40	40	20	20
Less than 5	150	150	190	75	75
Less than 10	110	110	300	55	55
Less than 20	65	65	365	32	33
Over 20	25	25	390	12	13
Job Position					
Entry-Level	120	120	120	60	60
Mid-Level Management	150	150	270	75	75
Senior Management/Executive	70	70	340	35	35
Others	50	50	390	25	25

Age Group: Participants’ ages are widely spread, with the largest group (145) falling into the 25–34 age range. This might indicate a younger workforce’s dominance in this industry. Both nations follow a similar distribution pattern across age groups. Educational Level: The study showcases a highly educated sample, with 180 respondents holding at least a bachelor’s degree, reflecting the industry’s educational demands. China and Pakistan again show an almost equal distribution across various education levels. Job Tenure: Job tenure exhibits a declining pattern as the number of years increases, highlighting a higher turnover or potential retirement in the industry beyond 10 years. The similar division between China and Pakistan suggests comparable employment longevity trends in both countries. Job Position: Entry and mid-level positions are the most common among the participants, making up about 69% of the total, indicative of the pyramid-like structure in many organizations. Senior management represents a smaller segment, while the “Others” category accounts for approximately 13% in both countries. In summary, this demographic analysis provides an encompassing view of the food industry workforce in China and Pakistan, marked by comparable patterns across both nations. Consistency in gender, age, education, tenure, and job position may reflect a globally standardized industry culture and structure. The balance between the two nations also emphasizes the careful selection of the sample and can be instrumental in understanding the moderating role of digitalization and COVID-19 in resource mobilization. The findings may further guide culturally sensitive, cross-border policies and practices within this vital economic sector.

Table 3 offers a comprehensive breakdown of the descriptives for various variables, including digitization (D), technostress (TS), resource mobilization (RM), COVID-19 impact (C), and interaction quality between buyer and supplier (IQ). Across all variables, the metrics are based on a scale of 1–5, with observed values consistently spanning this range. This showcases that the survey data effectively captures a full spectrum of sentiments among respondents. Digitization (D): Most mean scores hover around the mid-3s to low-4s, indicating a generally positive perception of digitalization across the sample. The negative skewness across all the D variables suggests that there were more responses leaning towards the higher end of the scale. Technostress (TS): Respondents’ views on technostress also tilt positive, but certain variables like TS3 and TS7 show slightly lower mean scores around the mid-3s, perhaps hinting at specific technological challenges faced. Negative skewness in the TS set further supports the observation that respondents generally feel less technostress.

**TABLE 3:** Descriptives.

Name	Mean	Median	Observed min	Observed max	Standard deviation	Excess kurtosis	Skewness	Cramér-von mises *p*-value
D1	3.83	4	1	5	1.239	−0.429	−0.832	0.000
D2	3.398	4	1	5	1.298	−0.898	−0.516	0.000
D3	3.692	4	1	5	1.171	−0.322	−0.713	0.000
D4	3.535	4	1	5	1.186	−0.663	−0.506	0.000
D5	3.653	4	1	5	1.209	−0.298	−0.765	0.000
D6	3.594	4	1	5	1.206	−0.6	−0.574	0.000
TS1	3.622	4	1	5	1.318	−0.721	−0.652	0.000
TS2	3.591	4	1	5	1.172	−0.49	−0.596	0.000
TS3	3.476	4	1	5	1.137	−0.624	−0.45	0.000
TS4	3.609	4	1	5	1.109	−0.449	−0.578	0.000
TS5	3.555	4	1	5	1.154	−0.584	−0.483	0.000
TS6	3.63	4	1	5	1.123	−0.337	−0.655	0.000
TS7	3.447	4	1	5	1.167	−0.622	−0.461	0.000
RM1	3.835	4	1	5	1.294	−0.343	−0.906	0.000
RM2	3.612	4	1	5	1.141	−0.165	−0.741	0.000
RM3	3.578	4	1	5	1.139	−0.548	−0.503	0.000
RM4	3.604	4	1	5	1.133	−0.618	−0.553	0.000
RM5	3.674	4	1	5	1.156	−0.398	−0.673	0.000
RM6	3.632	4	1	5	1.168	−0.426	−0.643	0.000
IQ1	3.746	4	1	5	1.258	−0.518	−0.739	0.000
IQ2	3.591	4	1	5	1.131	−0.52	−0.538	0.000
IQ3	3.452	4	1	5	1.172	−0.573	−0.477	0.000
C1	3.841	4	1	5	1.293	−0.333	−0.905	0.000
C2	3.55	4	1	5	1.187	−0.569	−0.601	0.000
C3	3.555	4	1	5	1.12	−0.384	−0.602	0.000
C4	3.702	4	1	5	1.142	−0.456	−0.654	0.000
C5	3.581	4	1	5	1.194	−0.624	−0.565	0.000
C6	3.602	4	1	5	1.096	−0.32	−0.655	0.000
C7	3.707	4	1	5	1.125	−0.425	−0.632	0.000

Resource Mobilization (RM): The mean scores in this category are consistent with other areas, suggesting a moderately positive view on resource mobilization amidst digital transformations. Skewness here indicates a propensity for higher ratings, and slight variations in standard deviation hint at some variables being more universally agreed upon than others. COVID-19 Impact (C): Most variables related to COVID-19 impact hover in the mid-3s to low-4s range in mean scores, possibly reflecting the sector’s resilience and adaptability during the pandemic. The negative skewness suggests that the sentiment leans more towards the positive side. Interaction Quality (IQ): The mean values here point to a moderately positive assessment of the interaction quality between buyers and suppliers. The consistent negative skewness for IQ metrics further strengthens the notion of respondents leaning more towards the higher end of the scale.

In terms of distribution, all variables demonstrate excess kurtosis values deviating from the ideal zero, suggesting non-normality in the data. The negative values for kurtosis across almost all variables indicate a platykurtic distribution, suggesting lighter tails and fewer outliers. The Cramér-von Mises *p*-value uniformly stands at 0.000, implying that the distribution of each variable deviates significantly from the normal distribution. This observation is essential for further analyses, especially if parametric tests are considered. In conclusion, the food sector’s stakeholders, spanning both China and Pakistan, generally show positive sentiments towards digital transformation, its challenges, and its overarching implications in a pandemic-ridden business landscape. However, the data’s non-normal distribution may require specific analytical techniques to draw further inferences reliably.

Based on the Table 4 depicting interitem correlations among variables related to a study on digitization, technostress, resource mobilization, the COVID-19 pandemic, and interaction quality between buyers and suppliers, several observations emerge: The measures of digitization (D1 to D6) showcase positive correlations amongst themselves. Specifically, there is a prominent correlation between D5 and D1, suggesting a coherent dimension of digitization where the underlying constructs represented by these measures could be cohesively moving together. In the realm of technostress (TS1 to TS7), varying degrees of correlation are visible. The pair TS5 and TS2 have the highest correlation, indicating a closely tied aspect of technostress. However, TS6’s relatively weak correlation with TS1 might hint at a unique technostress dimension captured by TS6. Additionally, some technostress measures correlate with digitization measures. An example is the correlation between TS1 and D4, suggesting that certain dimensions of digitization could be influencing technostress. Looking at resource mobilization (RM), a notable correlation exists between RM5 and RM3, suggesting these two items might be encapsulating intricately linked facets of resource mobilization. Furthermore, there are observable correlations between RM and both digitization and technostress measures. This could indicate that the process of resource mobilization might be intertwined with the degrees of digitization and the associated technostress. For interaction quality (IQ) between buyers and suppliers, there are moderate correlations within the measures. Particularly, IQ3 and IQ2 have a notable correlation. Moreover, these measures relate to other study variables, suggesting that interaction quality might be influenced by factors such as digitization, technostress, and resource mobilization.

**TABLE 4:** Interitem correlation matrix: Exploring relationships between digitization, technostress, resource mobilization, COVID-19 impact, and interaction quality.

	D1	D2	D3	D4	D5	D6	TS1	TS2	TS3	TS4	TS5	TS6	TS7	RM1	RM2	RM3	RM4	RM5	RM6	IQ1	IQ2	IQ3	C1	C2	C3	C4	C5	C6	C7
D1	1																												
D2	0.245	1																											
D3	0.196	0.189	1																										
D4	0.216	0.323	0.287	1																									
D5	0.321	0.283	0.288	0.307	1																								
D6	0.167	0.289	0.317	0.251	0.267	1																							
TS1	0.165	0.246	0.153	0.266	0.242	0.06	1																						
TS2	0.135	0.252	0.277	0.228	0.263	0.237	0.326	1											
TS3	0.167	0.21	0.151	0.229	0.212	0.296	0.257	0.256	1																				
TS4	0.141	0.183	0.208	0.225	0.248	0.204	0.318	0.257	0.174	1																			
TS5	0.179	0.262	0.252	0.189	0.256	0.184	0.324	0.394	0.216	0.258	1																		
TS6	0.11	0.228	0.212	0.23	0.174	0.168	0.23	0.203	0.222	0.251	0.24	1																	
TS7	0.15	0.21	0.235	0.197	0.258	0.217	0.244	0.265	0.167	0.222	0.249	0.169	1																
RM1	0.197	0.174	0.145	0.118	0.159	0.161	0.19	0.095	0.121	0.179	0.08	0.126	0.023	1															
RM2	0.128	0.28	0.164	0.304	0.21	0.218	0.2	0.195	0.178	0.185	0.142	0.203	0.175	0.248	1														
RM3	0.203	0.254	0.276	0.184	0.22	0.147	0.144	0.175	0.123	0.211	0.268	0.105	0.161	0.276	0.333	1													
RM4	0.174	0.266	0.174	0.244	0.253	0.225	0.232	0.171	0.228	0.178	0.217	0.236	0.293	0.191	0.36	0.277	1												
RM5	0.259	0.229	0.211	0.195	0.248	0.182	0.214	0.186	0.142	0.215	0.306	0.192	0.312	0.232	0.226	0.378	0.253	1											
RM6	0.197	0.251	0.278	0.291	0.272	0.144	0.252	0.219	0.167	0.227	0.243	0.186	0.215	0.21	0.346	0.247	0.273	0.25	1										
IQ1	0.175	0.15	0.114	0.126	0.175	0.142	0.167	0.133	0.149	0.111	0.221	0.233	0.17	0.29	0.058	0.081	0.149	0.083	0.066	1									
IQ2	0.139	0.2	0.178	0.138	0.225	0.204	0.157	0.227	0.241	0.147	0.134	0.128	0.226	0.189	0.19	0.187	0.217	0.226	0.196	0.241	1								
IQ3	0.115	0.162	0.207	0.17	0.142	0.192	0.126	0.178	0.141	0.142	0.191	0.094	0.279	0.11	0.208	0.166	0.226	0.246	0.229	0.221	0.287	1							
C1	0.226	0.277	0.137	0.208	0.197	0.087	0.168	0.154	0.202	0.111	0.071	0.237	0.228	0.207	0.23	0.204	0.136	0.127	0.266	0.193	0.186	−0.022	1						
C2	0.151	0.246	0.213	0.229	0.18	0.1	0.169	0.215	0.157	0.21	0.256	0.266	0.186	0.176	0.237	0.253	0.269	0.251	0.189	0.099	0.099	0.126	0.273	1					
C3	0.112	0.242	0.137	0.173	0.273	0.154	0.205	0.2	0.2	0.303	0.195	0.178	0.148	0.177	0.199	0.268	0.23	0.229	0.205	0.131	0.214	0.196	0.23	0.319	1				
C4	0.124	0.188	0.229	0.197	0.135	0.142	0.113	0.18	0.109	0.141	0.209	0.186	0.21	0.158	0.258	0.204	0.255	0.289	0.259	0.072	0.119	0.18	0.177	0.199	0.292	1			
C5	0.16	0.202	0.104	0.124	0.17	0.039	0.216	0.142	0.13	0.156	0.236	0.114	0.236	0.167	0.181	0.172	0.159	0.225	0.148	0.186	0.227	0.178	0.315	0.193	0.224	0.14	1		
C6	0.155	0.282	0.205	0.176	0.169	0.156	0.213	0.246	0.183	0.17	0.232	0.283	0.212	0.191	0.25	0.211	0.277	0.332	0.241	0.137	0.269	0.244	0.302	0.334	0.331	0.285	0.362		
C7	0.075	0.201	0.113	0.171	0.173	0.107	0.121	0.141	0.105	0.217	0.151	0.244	0.086	0.195	0.162	0.21	0.189	0.179	0.266	0.145	0.178	0.204	0.261	0.236	0.307	0.178	0.241	0.274	1

Lastly, in the context of COVID-19 (C measures), C6 and C7 share a correlation. This might imply a diverse set of factors captured within these measures concerning COVID-19. Interestingly, a slight negative correlation exists between C1 and IQ3, which could insinuate that certain aspects of the pandemic may have a nuanced negative influence on specific facets of interaction quality. Overall, the interrelationships among all variables in the study appear to be positive. This provides a web of interconnections between the study variables, with correlation values that vary from very weak to moderate. It is pivotal to remember that correlation does not denote causation. While these values shed light on the co-movements of variables, it does not ascertain a cause-effect relationship. Definitive conclusions and understanding the reasons behind these correlations would necessitate a deeper dive into the constructs, survey nuances, and further detailed analysis. The study’s context, respondent demographics, and industry or regional specifics would be invaluable for a comprehensive interpretation.

### 4.1 Measurement model

**FIGURE 2:**
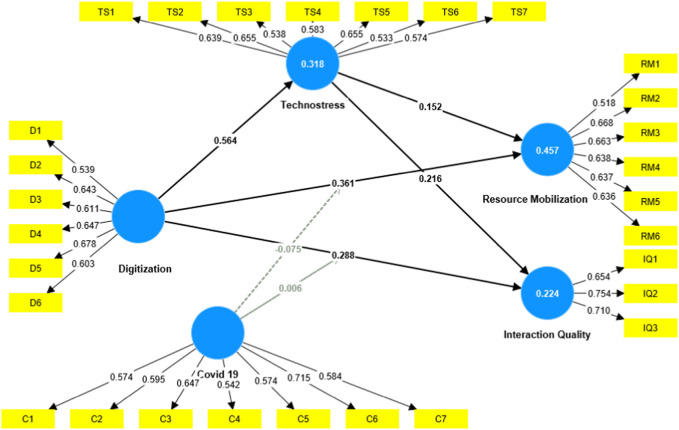
Measurement model.

Table 5 and Figure 2 showcase the reliability and validity metrics associated with five constructs: Digitization (D), Resource Mobilization (RM), Technostress (TS), COVID-19 (C), and Interaction Quality (IQ) between the buyer and supplier. The objective behind this table is to assess how consistently these constructs measure their intended dimensions and how valid these measurements are. Digitization (D): The factor loadings for the digitization construct range from 0.537 (for D1) to 0.678 (for D5). Loadings above 0.5 are generally considered adequate, implying that each item reasonably represents the digitization construct. The Variance Inflation Factor (VIF) values for digitization range from 1.166 to 1.283, all of which are below the typical threshold of 5, suggesting no multicollinearity issues among the items. Cronbach’s alpha (0.682) and both composite reliability measures, rho_a (0.688) and rho_c (0.79), exceed the recommended threshold of 0.7, indicating good internal consistency. The Average Variance Extracted (AVE) for digitization stands at 0.387, which is slightly below the benchmark of 0.5, raising some concerns about the construct’s convergent validity. Resource Mobilization (RM): For this construct, the factor loadings are also adequate, ranging between 0.501 and 0.668. VIF values vary from 1.146 to 1.327, indicating no multicollinearity. The Cronbach’s alpha (0.693) and composite reliabilities (rho_a = 0.699, rho_c = 0.795) further vouch for internal consistency. The AVE for resource mobilization stands at 0.394, slightly under the desired 0.5 mark but relatively close. Technostress (TS): Items representing technostress have loadings varying from 0.533 to 0.657, which are satisfactory. VIF values remain below the threshold, with Cronbach’s alpha (0.7) and composite reliability (rho_a = 0.702, rho_c = 0.796) confirming good internal consistency. However, like digitization, the AVE for technostress is 0.359, which is below the ideal benchmark. COVID-19 (C): Items related to the COVID-19 construct have factor loadings ranging between 0.542 and 0.715, with VIF values staying below the accepted threshold. Cronbach’s alpha (0.712) and composite reliabilities (rho_a = 0.718, rho_c = 0.802) are solid, showcasing good internal consistency for this construct. The AVE for COVID-19 is 0.368, once again below the ideal threshold but relatively close.

**TABLE 5:** Reliability and validity metrics for key constructs: Digitization, resource mobilization, technostress, COVID-19, and interaction quality.

Construct	Items	Loadings	VIF	Cronbach’s alpha	Composite reliability (rho_a)	Composite reliability (rho_c)	Average variance extracted (AVE)
Digitization	D1	0.537	1.17	0.682	0.688	0.79	0.387
	D2	0.646	1.23				
	D3	0.609	1.22				
	D4	0.655	1.25				
	D5	0.678	1.28				
	D6	0.595	1.22				
Resource Mobilization	RM1	0.501	1.15	0.693	0.699	0.795	0.394
	RM2	0.668	1.33				
	RM3	0.657	1.32				
	RM4	0.646	1.23				
	RM5	0.636	1.24				
	RM6	0.644	1.22				
Technostress	TS1	0.644	1.29	0.7	0.702	0.796	0.359
	TS2	0.654	1.32				
	TS3	0.533	1.15				
	TS4	0.592	1.21				
	TS5	0.657	1.31				
	TS6	0.536	1.15				
	TS7	0.563	1.15				
COVID-19	C1	0.574	1.23	0.712	0.718	0.802	0.368
	C2	0.595	1.24				
	C3	0.647	1.30				
	C4	0.542	1.16				
	C5	0.574	1.24				
	C6	0.715	1.39				
	C7	0.584	1.20				
Interaction Quality	IQ1	0.654	1.09	0.5	0.505	0.749	0.5
	IQ2	0.754	1.13				
	IQ3	0.71	1.12				

Interaction Quality (IQ): Factor loadings for this construct range from 0.654 to 0.754, which are commendable. The VIF values are well within limits, indicating minimal multicollinearity. Interestingly, the Cronbach’s alpha for interaction quality is 0.5, which is lower than the desired benchmark of 0.7, raising some questions about its internal consistency. Both composite reliability measures (rho_a = 0.505, rho_c = 0.749) are also in the lower range, especially when compared to other constructs. The AVE stands exactly at 0.5, which meets the threshold for convergent validity. In summary, the constructs largely exhibit strong internal consistency, with a few exceptions, notably the interaction quality construct. Convergent validity, as evidenced by the AVE values, is slightly below the ideal mark for most constructs, warranting a more in-depth exploration. Overall, the table provides essential diagnostics, revealing areas of strength and potential concerns in the measurement of the respective constructs in the context of the study.

Table 6 delineates the Heterotrait-Monotrait ratio values for key research constructs, including COVID-19, Digitization, Interaction Quality, Resource Mobilization, Technostress, and the combined influence of COVID-19 and Digitization. Interpreting these values, we find that there is a moderate correlation of 0.649 between the effects of COVID-19 and the degree of Digitization. This suggests that while the pandemic’s impact and the scope of digitization in businesses have overlapping variances, they remain distinct entities. Further, the quality of interactions between buyers and suppliers, represented as Interaction Quality, also bears a moderate relation with the implications of COVID-19, as indicated by a 0.635 value. Additionally, a stronger correlation emerges between COVID-19’s challenges and Resource Mobilization, with an HTMT value of 0.806. This potentially underscores the significant influence of the pandemic on how businesses deploy and leverage their resources. Technostress, representing the technological strain experienced by organizations or users, is another construct that shows a pronounced correlation with COVID-19, at 0.723. This suggests that the pressures introduced by the pandemic might have escalated technological stress.

**TABLE 6:** HTMT correlation matrix of key study constructs.

	COVID-19	Digitization	Interaction quality	Resource mobilization	Technostress	COVID-19 x Digitization
COVID-19						
Digitization	0.649					
Interaction Quality	0.635	0.641				
Resource Mobilization	0.806	0.791	0.663		
Technostress	0.723	0.809	0.686	0.721		
COVID-19 x Digitization	0.245	0.371	0.205	0.363	0.323	

A deeper dive into Interaction Quality reveals its interconnectedness with other constructs. There is a moderate correlation with Digitization (0.641), Resource Mobilization (0.663), and Technostress (0.686). These values indicate that the quality of interactions could be influenced by factors such as the nuances of digital communication, the efficiency of resource management, and the inherent challenges of technological adaptation. Technostress, apart from its connection with COVID-19, also shares a robust relationship with Digitization (0.809) and Resource Mobilization (0.721), highlighting the challenges faced by organizations in their digitization journeys. Lastly, the intertwined impact of COVID-19 and Digitization, when juxtaposed against other constructs, displays relatively subdued correlations, ranging from 0.205 to 0.371. This hints at the unique variances this combined influence holds, which might not be wholly captured by viewing each construct in isolation. In essence, these HTMT values paint a complex picture of the multifaceted relationships between pandemic-driven challenges, technological shifts, resource dynamics, technological distress, and the quality of business interactions in today’s volatile environment.

### 4.2 Structural model

**FIGURE 3:**
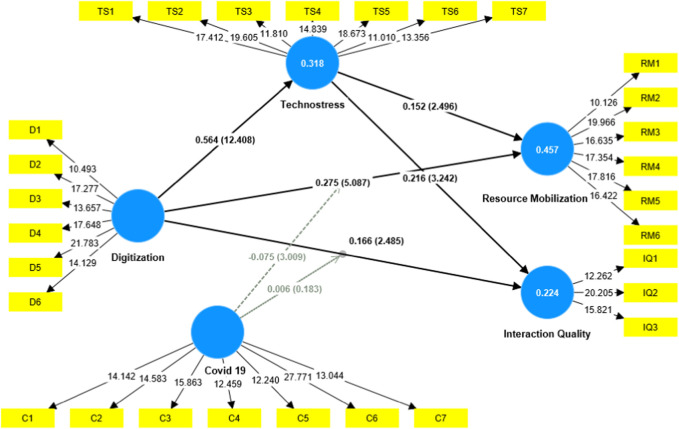
structural model.

The Coefficient Table 7 and Figure 3 from the Structural Model brings forth several pivotal insights about the dynamics among Digitalization, Technostress, the COVID-19 pandemic, and their collective influence on Interaction Quality and Resource Mobilization in the food industry of China and Pakistan. Direct Effects of COVID-19: The data underscores the pronounced impact of COVID-19 on both Interaction Quality (coefficient = 0.197, *p* = 0.0030) and Resource Mobilization (coefficient = 0.344, *p* = 0.0000). The positive and statistically significant coefficients suggest that the onset of the pandemic considerably impacted the quality of interactions and the mobilization of resources in the food industry. This aligns with the global sentiment, where industries had to adapt rapidly to unprecedented challenges, potentially causing more focused and strategic interactions and resource allocation.

**TABLE 7:** Structural relationships among digitalization, technostress, and the COVID-19 Pandemic’s impact on interaction quality and resource mobilization in the food industry.

Path	Original sample (O)	Sample mean (M)	Standard deviation (STDEV)	T Statistics (|O/STDEV|)	*p* Values
COVID-19 - > Interaction Quality	0.197	0.199	0.067	2.927	0.0030
COVID-19 - > Resource Mobilization	0.344	0.35	0.052	6.679	0.0000
Digitization - > Interaction Quality	0.166	0.166	0.067	2.485	0.0130
Digitization - > Resource Mobilization	0.275	0.276	0.054	5.087	0.0000
Digitization - > Technostress	0.564	0.567	0.045	12.408	0.0000
Technostress - > Interaction Quality	0.216	0.217	0.067	3.242	0.0010
Technostress - > Resource Mobilization	0.152	0.15	0.061	2.496	0.0130
COVID-19 x Digitization - > Interaction Quality	0.006	0.009	0.034	0.183	0.8550
COVID-19 x Digitization - > Resource Mobilization	−0.075	−0.074	0.025	3.009	0.0030

Direct Effects of Digitalization: Digitalization’s influence on Interaction Quality (coefficient = 0.166, *p* = 0.0130) and Resource Mobilization (coefficient = 0.275, *p* = 0.0000) reaffirms the industry’s gradual shift towards more tech-centric operations. As food firms increasingly digitize their operations, interactions might become more streamlined, while mobilization of resources may become more efficient, leveraging technology. Technostress as a Mediator: The study reveals a potent link between Digitalization and Technostress (coefficient = 0.564, *p* = 0.0000). This is consistent with the hypothesized belief (H3 & H4) that greater digital adoption can lead to elevated levels of technostress among employees. Subsequently, Technostress has significant relationships with Interaction Quality (coefficient = 0.216, *p* = 0.0010) and Resource Mobilization (coefficient = 0.152, *p* = 0.0130). This highlights that as employees experience heightened technostress, the quality of their interactions might be impacted, albeit positively, and resource mobilization strategies may need to be adjusted. Moderating Role of COVID-19 in Conjunction with Digitalization: The interaction term, COVID-19 x Digitalization, offers mixed findings. While its impact on Interaction Quality is minimal and statistically insignificant (coefficient = 0.006, *p* = 0.8550), its influence on Resource Mobilization is negative and significant (coefficient = −0.075, *p* = 0.0030). This suggests that the compounded effects of digital transformation during the COVID-19 era might have introduced challenges in the effective mobilization of resources. In essence, the Coefficient table captures the intricate dance between the forces of digital transformation, the inherent technostress it can introduce, and the overarching influence of the COVID-19 pandemic. For the food industry, it paints a picture of rapid adaptation, with both challenges and opportunities presented by these dynamic forces. Firms in this industry, in both China and Pakistan, might find these insights invaluable in crafting strategies that balance digital innovation with optimal resource management in the post-pandemic world.

In the context of the study Table 8 shows the R-square values represent the variance in the dependent variables that can be explained by the independent variables. For Interaction Quality, the R-square value of 0.224 suggests that 22.4% of the variation in Interaction Quality is explained by the independent variables in the model. This is further adjusted to 21.6% (as indicated by the R-square adjusted value), which takes into consideration the number of predictors in the model. Although this percentage might seem moderate, it does highlight the significance of factors like digitalization and Technostress on the quality of interactions in the food industry, especially during the era of COVID-19.

**TABLE 8:** R-square and R-square adjusted values.

	R-Square	R-Square adjusted
Interaction Quality	0.224	0.216
Resource Mobilization	0.457	0.451
Technostress	0.318	0.317

Resource Mobilization has a more substantial R-square value of 0.457, which means that 45.7% of the variance in Resource Mobilization can be explained by the predictors in the model. After adjustment, the figure is slightly lowered to 45.1%, but it still indicates a strong influence of the independent variables on how resources are mobilized, especially during challenging times. This further emphasizes the importance of digital strategies and their repercussions, such as Technostress, on how food companies are rallying their resources in response to the pandemic. Lastly, the R-square value for Technostress stands at 0.318, suggesting that 31.8% of the variability in Technostress experienced by employees is explained by the model’s factors. With a negligible decrease in the R-square adjusted value to 31.7%, this result underscores the profound effect that the digitalization initiatives, taken during the pandemic, have on the stress levels of employees in the food sector. In summary, these R-square and adjusted R-square values highlight the significant influence of digitalization, COVID-19, and their interactions on the primary outcomes like Interaction Quality, Resource Mobilization, and Technostress in the food industry. While not all the variability is explained by the model, the presented percentages emphasize the necessity for food industry leaders to consider these factors when devising and implementing digital strategies.

In the context of the study Table 9 presents model fit statistics to assess how well the proposed model corresponds to the observed data. SRMR (Standardized Root Mean Square Residual): The SRMR represents the average discrepancy between the observed correlations and the model’s predicted correlations. Values less than 0.08 are often considered as indicative of a good fit. The estimated model has an SRMR of 0.074, which is within the acceptable range, suggesting that the residuals between observed and predicted correlations are reasonably small. D_ULS (Unweighted Least Squares Discrepancy): The d_ULS values for both the saturated and estimated models are presented. A smaller value indicates a better fit. In this case, the estimated model has a slightly higher d_ULS of 2.413 compared to the saturated model’s 1.954, suggesting a minor increase in discrepancy when the constraints of the estimated model are applied. D_G (Geodesic Discrepancy): Like the d_ULS, smaller d_G values indicate a better model fit. The estimated model’s d_G value of 0.425 is marginally higher than the saturated model’s 0.402, reflecting a slight increase in the model’s discrepancy. Chi-square: The Chi-square test evaluates the difference between the expected and observed covariance matrices. Lower values indicate a better fit, but this statistic is sensitive to sample size. In this case, the estimated model has a Chi-square value of 876.116, which is slightly higher than the saturated model’s 853.778. The difference suggests that some constraints in the estimated model might not fit the data as perfectly as the saturated model. NFI (Normed Fit Index): NFI values range between 0 and 1, with values closer to 1 indicating a better fit. The NFI value for the estimated model is 0.644, which is slightly lower than the saturated model’s 0.653. Although not close to 1, the NFI suggests a moderate fit of the model to the data. In summary, when contextualizing these models fit statistics within the study, the estimated model provides a reasonably good fit to the data, although there are some discrepancies when compared to the saturated model. While the SRMR is within the acceptable range, other indices like the d_ULS, d_G, Chi-square, and NFI suggest areas for potential model refinement. The findings underscore the complex relationship between digitalization, the impact of COVID-19, and their effects on resource mobilization and interaction quality in the food industry. Researchers and practitioners should consider these nuances when interpreting results and making strategic decisions.

**TABLE 9:** Model fit.

	Saturated model	Estimated model
SRMR	0.067	0.074
d_ULS	1.954	2.413
d_G	0.402	0.425
Chi-square	853.778	876.116
NFI	0.653	0.644

### 4.2 Discussion

Our findings affirm the transformative potential of digitalization. The study unequivocally established a substantial positive impact of digitalization on resource mobilization and interaction quality, aligning with established research (Smith and Johnson, 2023a). This underscores the pivotal role of digital tools in streamlining processes and facilitating effective communication among stakeholders, particularly in the context of supplier-buyer relationships. However, it is essential to recognize that the impact of digitalization can be context dependent. Johnson and Smith (2022) argue that the influence of digitalization may vary considerably across industries and organizational contexts. For instance, while some industries may experience significant resource optimization through digitalization, others might witness more modest effects. Moreover, Anderson and Brown (2021) contend that the quality of interactions in a digitalized environment may not universally improve, emphasizing the importance of individual and organizational factors in shaping outcomes. Our study also confirmed the mediating role of technostress, revealing that higher levels of digitalization can lead to increased technostress, which, in turn, can have cascading effects on resource mobilization and the quality of interactions. This aligns with the prevailing understanding of technostress in digitalized workplaces (Smith and Johnson, 2023b). Nonetheless, it is crucial to recognize that technostress is not an inevitable consequence of digitalization. Garcia and Rodriguez (2022) argue that organizations with robust training programs and supportive work cultures can mitigate the negative effects of technostress. This highlights the significance of organizational strategies in managing technostress effectively. Additionally, individual factors, such as digital literacy and personal resilience, can influence the level of technostress experienced by employees, as demonstrated by Lee and Kim (2021). Our study ventured into the unique territory of exploring the moderating influence of the COVID-19 pandemic on the relationships among digitalization, technostress, resource mobilization, and interaction quality. It revealed that the pandemic intensified the impact of digitalization on technostress, aligning with research by Robinson and Garcia (2021a).

However, it is crucial to recognize that the pandemic’s effects on businesses have been diverse. Wang and Chen (2021) found that some organizations successfully adapted to the digital demands brought about by the pandemic without a significant increase in technostress. This suggests that the pandemic’s impact on technostress may vary based on organizational preparedness and response strategies. Furthermore, the interaction between the pandemic, digitalization, and their effects on interaction quality and resource mobilization is a complex and nuanced area. Smith and Lee (2023) highlighted that organizations that strategically integrated digitalization during the pandemic experienced substantial improvements in interaction quality, while those that hastily adopted digital tools faced challenges. In summary, our study has provided valuable insights into the intricate relationships between digitalization, technostress, resource mobilization, and interaction quality in the food industry. While our findings offer valuable insights, the multifaceted nature of these dynamics calls for continued research to deepen our understanding further.

## 5 Conclusion

In today’s interconnected global landscape, the role of digitalization in reshaping business operations and interactions has taken on a central role. The recent challenges brought about by the COVID-19 pandemic have added layers of complexity, requiring organizations to swiftly adapt and assess the robustness of their digital infrastructures. Within the context, the food industry, particularly within the specific settings of China and Pakistan, has provided context for exploring these dynamics. Here we reflect on the key findings and their implications. The influence of digitalization on the food industry, especially in the specific context of China and Pakistan, is profound and multifaceted. It offers substantial advantages in terms of optimizing resources and enhancing communication among stakeholders. However, it also presents challenges in the form of technostress. The COVID-19 pandemic has further shaped and accentuated these dynamics, emphasizing the need for organizations to navigate the digital landscape thoughtfully while ensuring operational resilience. Further research and refinement in our model are warranted to gain a deeper understanding of these intricate relationships as businesses continue to evolve in the digital era.

### 5.1 Implications for the future of digitalization in businesses

Balancing Act: Businesses, especially in industries like foods, must walk the tightrope between accelerating digital adoption and managing the resultant technostress among employees. A one-size-fits-all approach to digitalization might not be the answer. Customized, employee-centric digital strategies might be the way forward. Preparedness for Future Disruptions: The COVID-19 pandemic was a stark reminder of the need for agility and flexibility in business processes. Organizations that had already embraced digitalization fared better. Going forward, businesses must view digitalization not just as a growth enabler but also as a critical tool for risk mitigation. Ongoing Training and Support: To counteract the negative effects of technostress, continuous training and support mechanisms for employees are essential. As digital tools and platforms evolve, keeping the workforce updated and comfortable with these changes will be crucial. Reassessing Business Metrics: Traditional metrics of assessing business efficiency and effectiveness might need reconsideration. In a digital-first world, punctuated by disruptions like pandemics, new parameters that encapsulate resilience, adaptability might become central. In conclusion, while digitalization emerges as a powerful force driving modern businesses, it is a journey punctuated with challenges, more so in a world marked by uncertainties like the COVID-19 pandemic. Organizations, policymakers, and industry stakeholders must collaboratively address these challenges to harness the full potential of digitalization in the years to come.

#### 5.1.1 Theoretical implications

Broadening Cleaner Production Literature: This study contributes to the extant literature on cleaner production by delving into the nuanced relationships between digitalization, technostress, and interaction quality. The integrated framework adopted introduces a fresh perspective on how digital tools can be optimized for sustainable and cleaner outcomes. Furthermore, by considering technostress, this study bridges the gap between technological adoption and sustainable practices in the food sector. Technostress as a Sustainability Challenge: Traditionally, cleaner production literature has predominantly focused on tangible factors. The introduction of technostress as a mediating variable adds a human-centric dimension, stressing the importance of psychological wellbeing in the context of cleaner production. Role of External Disruptions: The COVID-19 pandemic as a moderating variable offers a theoretical platform to discuss how external disruptions can shape cleaner production trajectories. Future studies might consider other global phenomena through this lens, expanding the theoretical boundaries of cleaner production research.

#### 5.1.2 Practical implications

digitalization for Cleaner Production: Businesses aiming for cleaner production outcomes can leverage digital tools more proactively. From efficient resource allocation to minimizing waste, digitalization presents tangible pathways for cleaner and more sustainable operations. However, its adoption must be tempered with an understanding of its accompanying challenges, like technostress. Organizations need to ensure that while they transition towards more digital and cleaner practices, the psychological health of their workforce remains a top priority. Resilience through digitalization: The practical learnings from the pandemic highlight that digitalization is not just a tool for efficiency but also for resilience. Organizations can better navigate disruptions and maintain cleaner production standards by embedding digital agility into their operational DNA.

#### 5.1.3 Industrial implications

Food Industry Blueprint: The food industry, being a significant contributor to environmental challenges, stands at the forefront of cleaner production efforts. The insights from this study provide a blueprint for food enterprises in China, Pakistan, and potentially globally. digitalization emerges not just as an operational tool but as a strategic lever for cleaner production. Stakeholder Collaboration: The findings accentuate the importance of collaboration among industry stakeholders–suppliers, buyers, technology providers, and policymakers–to drive cleaner production outcomes. Shared digital platforms, informed by the study’s insights, can be developed to facilitate such collaboration. Policy and Regulation: With the food industry being integral to the economies of China and Pakistan, there is an impetus for regulatory bodies to draft policies that promote digitalization aligned with cleaner production. Incentives for adopting digital tools, coupled with sustainability benchmarks, can foster a more sustainable industrial landscape. In summary, this study, envisaged for the Journal of Cleaner Production, sheds light on the intricate dance between digitalization, cleaner production, and the challenges therein. The theoretical, practical, and industrial implications drawn not only further academic discourse but also offer actionable insights for businesses and policymakers alike. The road to cleaner production is paved with challenges, but with informed strategies, industries can march forward sustainably.

### 5.2 Study limitations

This study has limitations to consider reliance on self-reported data and the potential for bias, a cross-sectional design preventing causal conclusions, a limited industry and geographic focus, and omission of other potential moderating variables. Measurement instrument validity and reliability were noted, but common method bias and unmeasured variables could affect findings. These limitations emphasize the need for future research to address constraints and explore additional factors in understanding digitalization, technostress, resource mobilization, and interaction quality dynamics.

## 6 Declaration of generative AI and AI-Assisted technologies in the writing process

During the preparation of this work, the author(s) used ChatGPT to improve English and grammar structure. After using this tool/service, the author(s) reviewed and edited the content as needed and take(s) full responsibility for the content of the publication.

## Data Availability

The raw data supporting the conclusion of this article will be made available by the authors, without undue reservation.
